# *In-Vitro* Visualization of Thrombus Growth in Artificial Lungs Using Real-Time X-Ray Imaging: A Feasibility Study

**DOI:** 10.1007/s13239-021-00579-y

**Published:** 2021-09-16

**Authors:** Andreas Kaesler, Freya Lilli Rudawski, Mark Oliver Zander, Felix Hesselmann, Isaac Pinar, Thomas Schmitz-Rode, Jutta Arens, Ulrich Steinseifer, Johanna Charlotte Clauser

**Affiliations:** 1 Department of Cardiovascular Engineering, Institute of Applied Medical Engineering, Helmholtz Institute, Medical Faculty RWTH Aachen University, Aachen, Germany; 2 Monash Institute of Medical Engineering and Department of Mechanical and Aerospace Engineering, Monash University, Melbourne, Australia; 3 Chair of Engineering Organ Support Technologies, Department of Biomechanical Engineering, Faculty of Engineering Technology, University of Twente, Enschede, The Netherlands

**Keywords:** Hemocompatibility, ECMO, Oxygenator thrombosis, Artificial surface, Phase-contrast X-ray imaging

## Abstract

**Purpose:**

Extracorporeal membrane oxygenation has gained increasing attention in the treatment of patients with acute and chronic cardiopulmonary and respiratory failure. However, clotting within the oxygenators or other components of the extracorporeal circuit remains a major complication that necessitates at least a device exchange and bears risks of adverse events for the patients. In order to better predict thrombus growth within oxygenators, we present an approach for *in-vitro* visualization of thrombus growth using real-time X-ray imaging.

**Methods:**

An *in-vitro* test setup was developed using low-dose anticoagulated ovine blood and allowing for thrombus growth within 4 h. The setup was installed in a custom-made X-ray setup that uses phase-contrast for imaging, thus providing enhanced soft-tissue contrast, which improves the differentiation between blood and potential thrombus growth. During experimentation, blood samples were drawn for the analysis of blood count, activated partial thromboplastin time and activated clotting time. Additionally, pressure and flow data was monitored and a full 360° X-ray scan was performed every 15 min.

**Results:**

Thrombus formation indicated by a pressure drop and changing blood parameters was monitored in all three test devices. Red and white thrombi (higher/lower attenuation, respectively) were successfully segmented in one set of X-ray images.

**Conclusion:**

We showed the feasibility of a new *in-vitro* method for real-time thrombus growth visualization by means of phase contrast X-ray imaging. In addition, with more blood parameters that are clinically relevant, this approach might contribute to improved oxygenator exchange protocols in the clinical routine.

## Introduction

Extracorporeal membrane oxygenation (ECMO) has gained increasing attention in the treatment of patients with acute and chronic cardiopulmonary and respiratory failure. However, coagulation disorders such as severe bleeding events and clot formation in the ECMO circuit are still unpredictable and may lead to critical incidents during extracorporeal life support (ECLS).^11^ In 2016, 36.8% of all complications were induced by clot incidents in adult ECLS treatment. Mechanical complications due to clot formation in the oxygenator alone accounted for 11.2% of total complications.^13^ These adverse events necessitate at least the replacement of the oxygenator in an ECMO circuit, indicated by reduced gas exchange performance or increased membrane pressure drop (dpMO).^20^ Furthermore, loss of platelets and fibrinogen, a decrease in activated clotting time (ACT) and activated partial thromboplastin time (aPTT) along with an increase in D-Dimers suggest progression of clot formation within membrane oxygenators during ECMO therapy.^10,11,16^

In clinical practice, the decision of replacing an oxygenator is mainly based on the experience of the medical team due to a lack of clearly defined thresholds of the mentioned technical parameters. Therefore, current efforts focus on the development of prediction models of membrane oxygenator thrombosis and therewith defined indicators to assist the medical staff to decide early on whether a device needs to be replaced.

Most studies have matched thrombus volume quantified after acute system exchange^9,12,22,25^ to earlier collected monitoring data. However, these results provide limited information on the effect of thrombus growth on monitoring parameters due to the discrete and retrospective character of quantification as well as potential interference of the weaning process. Another approach attempts to describe the direct effect of differently sized thrombi on continuous membrane oxygenator thrombosis monitoring parameters in *in-vitro* blood loops. In a previous study, we have demonstrated the decrease of CO_2_ exhaust gas concentration and moderate increase of dpMO as a response to different sizes of silicone volumes in oxygenators,^18^ whereas Krivitski *et al.* have successfully correlated ultrasonic measurements of effective oxygenator blood volume to the size of artificial soft paraffin thrombi.^20^ Both studies are limited by using simulated static thrombi, not taking the dynamics of thrombosis into account.

Furthermore, indicators such as dpMO increase or platelet drop are only indirect hints for an already formed clot and do not allow for an early-phase prediction of the onset of thrombus formation. Additionally, increasing dpMO was suspected to depend rather on thrombus location than size,^20^ which is supported by Lehle *et al.* stating that increasing dpMO is a rare device related indicator of MOT.^21^ Especially during the development and approval testing of oxygenators, observation of early-stage thrombus formation could provide essential information about the thrombotic potential of the device.

Due to the opacity of blood, the membrane fibers and parts of the oxygenator housing mapping of thrombus deposits requires advanced visual measurement techniques. A common method for flow field visualization in medical devices is particle image velocimetry (PIV). However, it is based on optical measurements that require a transparent device as well as transparent fluids. Thus, particle image velocimetry makes use of transparent blood analogue fluids^30^ or platelet rich plasma as blood derivate,^7^ which neglect the complex interactions of all blood components during thrombus formation. As alternative, imaging techniques such as computed tomography (CT) or plain radiography overcome the limitation of the blood’s opacity but most often require contrast agents or tracer particles for flow visualization, which can influence the clotting behavior, and have a lower spatial resolution.^1^ Few studies have attempted to combine CT technology with flow field visualization by using the inherent optical properties of red blood cells by means of synchrotron X-ray imaging^17^ and 4D computed tomography.^28^

In this study, we used phase-contrast X-ray imaging for the visualization of real-time thrombus growth in an oxygenator while measuring dpMO, platelet count, ACT and aPTT over the time course of the experiments. The aim was to establish a suitable method for thrombus growth detection in oxygenators that allows for the distinct correlation of clinical parameters with beginning oxygenator thrombosis in the future and thereby improving both oxygenator development and patient treatment.

## Materials and Methods

### Object of Study: The NeonatOx Oxygenator

As study object we choose the NeonatOx oxygenator, which was developed by Arens *et al.* to support neonates as a bridge to lung recovery option and has been tested thoroughly *in vitro* and *in vivo*.^2,29^ The gas exchange membrane consists of polypropylene fibers (OXYPHAN® Capillary membrane Type PP 50/200, 3M Membrana, Wuppertal, Germany) with an outer diameter of 300 *µ*m and an average gap between individual fibers of 260 *µ*m. For the purpose of this study, we manually manufactured three NeonatOx devices according to the same standard fabrication protocol; a detailed description of the manufacturing process and device properties can be found in previously published work.^2^ Briefly, blood enters the device through an inner core at the bottom and is guided towards the fiber bundle through a punctual transition from inlet geometry to fiber bundle and exits the device at the top. Compared to adults, neonates have less oxygen consumption, less cardiac output, and less blood volume, which enables the device to have a reduced number of hollow fiber membranes, a blood flow design optimized for low flow rates and a minimization of overall priming volume, respectively.

### Computed Tomography Setup

**Figure 1:**
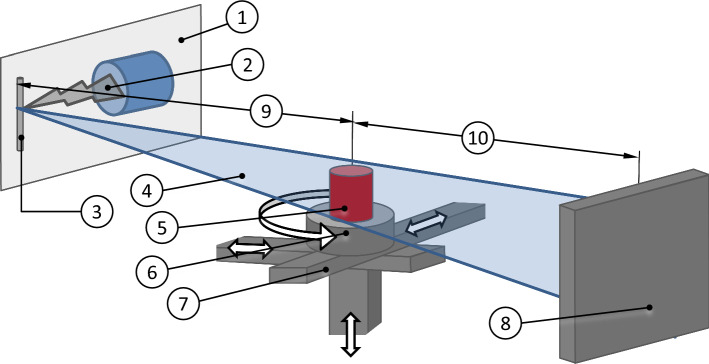
Schematic of CBCT set up used for imaging the NeonatOx: liquid metal-jet X-ray source (1), electron beam (2), liquid metal jet (Ga–In-alloy) anode (3), X-ray cone beam (4), object of interest/NeonatOx (5), rotational degrees of freedom (DOF) (6), translational DOF (7), flat panel detector (8), distance between source and NeonatOx of 750 mm (9), distance between NeonatOx and detector of 1445 mm (10).

Computed tomography (CT) scans were acquired via a custom laboratory X-ray setup, developed at Monash University (Clayton, Victoria, Australia), primarily mentioned in the context of micro *in-vivo* imaging in literature.^28^ The X-ray source is a liquid metal jet anode, which creates an X-ray cone beam with an effective aperture angle of 10.5° (MetalJet D2+ 70kV, Excillum AB, Kista/Sweden), see Fig. 1. The anode is comprised of a liquid metal jet from Gallium Indium alloy (ExAlloy G1, Excillum AB), with an X-ray wavelength of 1.34 Å. The cone beam computed tomography (CBCT) setup consists of a mobile low noise flat panel detector with a 1024 x 1024 pixel array and a pixel pitch of 194 *μ*m (PaxScan 2020, Varian Medical Systems, Palo Alto/CA/USA). A stage system with four degrees of freedom is utilized for rotating the NeonatOx device (T-RSW60A motorized rotary stage, Zaber Technologies Inc., Vancouver/BC/Canada), see Fig. 1. For CT imaging, the NeonatOx rotates 360° while the detector simultaneously records the energy absorption by the NeonatOx and processes the two-dimensional X-ray projections at various angles.

Instead of measuring the absorption of electromagnetic radiation, which is common in the clinical setting, we used phase contrast X-ray imaging, which detects a change in amplitude (attenuation) and phase as the X-ray beam passes through the study object and therefore captures the change in phase in real time.^24,31^ This approach provides enhanced soft-tissue contrast, which improves the differentiation between blood and potential thrombus growth.^27^ The effective brightness or X-ray source power density is modified by adjusting the power and the cross-sectional area of the electron beam hitting the liquid jet anode. Prior to the present study, we found that the highest image brightness and contrast of the X-ray projections was achieved by using a 60 *μ*m x 15 *μ*m beam spot size at a power load of 240 W. In order to increase the temporal resolution of monitoring potential thrombus growth between consecutive CT scans, it is desirable to generate short CT scan times. A short scan time can be achieved by increasing the rotation speed of the NeonatOx and image acquisition frequency; however, this would have led to loss in image brightness, contrast decrease and motion artefacts, such as feature blurring. For our study, a scan time of 12 min (0.50 s^−1^ rotation speed), with a total of 5000 projections per 360° at an image acquisition frequency of 7 fps was found to be the best compromise between temporal resolution and image quality. The ionization dose applied on the setup corresponds to a standard chest CT.

### *In-Vitro* Blood Loop

**Figure 2:**
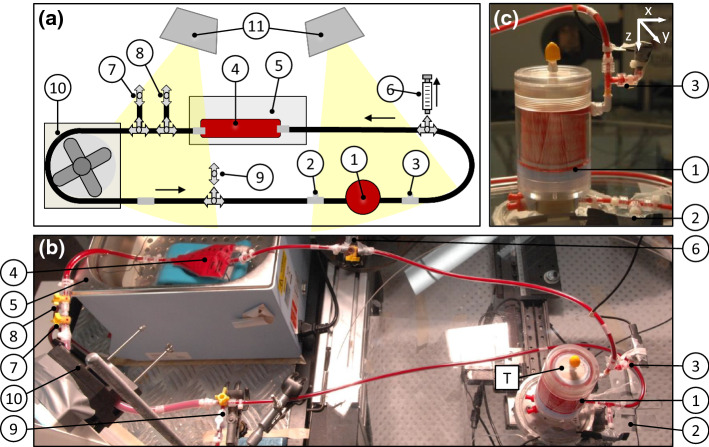
(a) Sketch and (b) photography of the blood loop, (c) close up photography of the NeonatOx oxygenator; 1: NeonatOx, 2: pressure sensor P1, 3: pressure sensor P2, 4: blood reservoir, 5: water bath, 6: blood sampling port, 7: blood filling port, 8: blood draining port, 9: air removal port, 10: roller pump, 11: heat lamps (500 W), T: position of IR temperature measurement at the cylindrical oxygenator housing top.

The *in-vitro* blood loop, presented in Fig. 2, was built as a closed loop to create controlled blood flow conditions inside the NeonatOx, while considering the spatial restrictions given by the CBCT setup, illustrated in Fig. 1. The thrombotic potential of other blood loop components was minimized by reducing tube lengths and avoiding possible blood flow obstructions, such as inline temperature, pressure or flow sensors. Main components were the NeonatOx oxygenator (1), a roller pump (Masterflex® L/S® Computerized Drive and Easy Load® II Head, Cole Parmer Instrument Co., Vernon Hills/IL/USA) (10) and a 50 mL blood reservoir (Enteral Drainage Bag, Thermo Fisher Scientific Inc. Corp.) (4) in a water bath (5), see Fig. 2. Pressure was measured pre- (2) and post-device (3) with two 30 psi absolute pressure sensors (TruStability®, Honeywell International Inc., Morris Plains/NJ/USA). The temperature was measured at the oxygenator housing via an IR Thermometer (IT 1 IR Thermometer, RS Components Ltd., Corby/UK), see Fig. 2b (T). Blood samples were taken post NeonatOx (6), while various other ports were used to fill (7) and drain (8) the blood loop, as well as to remove air bubbles (7-9). The components were connected via 1/8” inner diameter polyvinylchloride (PVC) tubing (Nordson Corp.) except for the roller pump tube which required a 1/4″ silicone tubing (Masterflex® L/S® 24, Masterflex SE, Germany). In addition to the water bath, two heat lamps (11) were installed to control temperature.

The blood contacting area of the flow loop resulted to 0.0356 m^2^ with a priming volume of 140 mL, whereas, the NeonatOx devices provide a membrane surface area of approximately 0.12 m^2^ and a priming volume up to 20 mL. Thereby, the exposure ratio of test surface area to blood results to 7.5 cm^2^ mL^−1^ and a test surface to foreign surface area ratio of 3.37. To allow for comparison with previous work,^2,29^ we choose to use a flow rate of 95 mL min^−1^ for all three experiments.

### Blood Collection and Preparation

Fresh ovine blood was collected from three healthy Border Leicester Merino crossbreed sheep from the Monash Animal Research Platform (MARP, Monash University, Clayton/Victoria/ Australia). All animals are kept in healthy conditions and are regularly subjected to blood sampling for research purposes, in accordance with MARP's Standard Operating Protocol for sheep blood donation. All blood samples were collected from the left jugular vein after the injection of 1 mL of a local anesthetic (Bupivacaine Injection 0.5%, Pfizer Australia Pty Ltd, West Ryde/New South Wales/ Australia). During sampling, the sheep were restrained in the standing position.

First, two blood samples were withdrawn for initial blood count into two 1 mL K_3_EDTA tubes (IDEXX VetCollect® tube EDTA, IDEXX Laboratories Pty. Ltd., Rydalmere/New South Wales/Australia) using a 21 G × 1″ BD Vacutainer® needle (Becton, Dickinson and Company, Franklin Lakes/NJ/USA). Subsequently, a 14 G × 50 mm catheter (Optiva® I.V. Catheter, Smiths Medical Inc., Minneapolis/MN/USA) was inserted into the left jugular vein. After discarding the initial drops of blood from the catheter, blood samples (in total 200–250 mL) were drawn into 50 mL syringes (Terumo Corp., Tokyo/Japan). The syringes were pre-loaded with unfractionated heparin (H3393, Sigma Aldrich, St. Louis/MO/USA) with a final concentration of 2.5 USP mL^−1^ blood.

Within 20 min after blood collection started, all blood samples were transported to our laboratories in a portable insulated container. From the start of transport until the final loading of the loop, all samples were gently mixed continuously. Upon arrival, all heparinized samples were rested for a minimum of 15 min at 37 °C based on studies on reversing temperature induced and transport-induced platelet activation.^23,32^ Blood temperature was monitored using IR surface thermometers.

The blood count (ProCyte Dx Haematology Analyser, IDEXX Laboratories Inc.) of the EDTA reference samples was tested after a resting period of 5 min (animal baseline). Heparinized blood samples were tested for blood count as well and results were compared to the animal baseline value. Syringes with less than 250,000 platelets *µ*L^−1^ and below 70% of animal baseline values were discarded for the experiment. The remaining syringes were pooled in a 450 mL blood bag (Teruflex® blood bag without anticoagulant, Terumo Corp.). After 5 min of gentle mixing at 37 °C, the blood count of the pooled blood was measured and defined as the new baseline value (blood bag baseline) for blood count testing during the experiment. An additional sample was drawn from the blood bag and stored at 37 °C as the static control.

### *In-Vitro* Experiments

First, the blood loop was primed with 140 mL NaCl, air bubbles were removed, and temperature was adjusted to 37 °C ± 1 °C via water bath and heat lamps. NaCl was circulated in the loop with 95 mL min^-1^ for a minimum of 90 min prior to test begin to warm up all components of the loop. In the following, the flow loop was opened, the blood bag was connected to one end and flow was slowly increased to 20 mL min^−1^, thereby displacing the NaCl from the system at the other end. After removal of all NaCl, the loop was connected to the blood bag and blood flow was slowly increased to the target value of 95 mL min^-1^. During the experiments, the blood loop surface temperature was monitored every 15 min at the oxygenator housing (T) marked in Fig. 2b. Blood samples were taken from the blood sampling port, see Fig. 2, at the beginning of the experiment, after 15 min and then every 30 min. A volume of 3 mL blood was drawn with a 21 G needle (Sterican® single use hypodermic needle, B.Braun Melsungen AG) connected to a 5 mL syringe (Terumo Corp.). Approximately 1 mL of blood was transferred into a pre-warmed EDTA tube and subjected to blood count analysis. The remaining blood was immediately tested for aPTT and ACT (Hemochron® Signature Elite System with aPTT test cuvette and ACT LR test cuvette—Celite®, Werfen Australia, Artarmon/New South Wales/Australia). The blood reservoir was manually mixed every 15 min for 1 min to minimize cell sedimentation. Blood was sampled from the loop before and after the first 360° CT scan (CT1), and afterwards blood sampling continued every two consecutive 360° full scans (CT2–CT8). Although the purpose of an oxygenator is gas transfer, in the present study we were only interested in the hemodynamics and the thrombus growth within the device; thus, the gas transfer performance was not investigated. Since flow and material induced thrombogenicity are the main reported causes of oxygenator membrane clotting, a possible influence of the gas exchange on thrombogenicity can be neglected ^11^.

For the experiment, several boundary conditions for success were defined: (i) experiments must conclude within a 4-h time frame from the moment of blood drawing from the animal, because of a decrease in blood vitality *in vitro*.^3,4^ (ii) Red blood cell count must be relatively stable for the duration of the experiment, because a decline in red blood cell count alters the X-ray attenuation coefficient of the free-flowing blood in the CT scans. This would complicate the comparisons of consecutive CT scans, because no additional contrast agent besides hemoglobin was used. (iii) Severe coagulation indicated by the absence of platelets in the blood samples, as well as a rapid increase in dpMO terminates the experiment.

### X-ray Image Processing

Image processing was performed with the software X-TRACT (Commonwealth Scientific and Industrial Research Organisation, Canberra/Australia),^15^ using flat field correction, ring filters and error-based correlation to correct for detector inherent artefacts during pre-processing of 2D projections (5000) and the default linear ramp filter for the TJD-FDK back-projection^14^ during slice (1024) reconstruction. All slices of one CT scan (1024 slices) were stacked in the vertical (axial) direction for post-processing including volume rendering and material segmentation^8^ with the software Amira Avizo 3D (Thermo Fisher Scientific Inc. Corp., Waltham/MA/USA).

### X-ray 3D Image Reconstruction

Instead of determining the exact attenuation coefficients for white and red clots, the first CT scan (CT1) was used as a baseline. CT1 was performed immediately after the first blood sampling, see Fig. 8, when we can assume that thrombotic deposition has not yet occurred, and that the images therefore show the attenuation of free-flowing whole blood. The final CT scan (CT8) was subtracted from CT1 based on manual single frame subtraction, as the rotation of the study object was not triggered. The outer device housing edges were used to ensure an image alignment within the accuracy of ±1 pixel.

The concentration of red blood cells (RBCs) in red and white clots results in a higher and lower intensity change in comparison to free-flowing whole blood, respectively.^5,6,19^ Therefore, higher, and lower intensity change in the subtraction images in reference to CT1 indicates areas of potential red and white thrombotic deposition, respectively.

## Results

The experiments were performed on three NeonatOx devices (Neo1–Neo3). Neo1 showed severe coagulation within the first 15 min of the experiment. Neo2 and Neo3 showed a severe thrombotic event within 90 and 110 min after the start of the experiment, respectively. In the following, we present blood, pressure and temperature data from all three experiments, but have decided to focus on image analysis of Neo2 only, which we consider one of two successful runs in terms of blood management. We believe that image data of one device is sufficient to discuss whether our newly introduced method for monitoring thrombus growth with phase-contrast X-ray imaging in real-time is feasible.

### Visual Inspection

Oxygenators and the test loops were visually examined with regard to adherent thrombi or other blood residues after flushing. Thrombi were found in the oxygenators at the overlapping fiber mat edge, in the outlet region and the lower blood compartment area. Moreover, thrombi adhered to the tubing connected to the pressure sensors, some connectors and in the blood bag.

### Blood and Coagulation Parameter

The blood count values differed for each animal and thus each test day; therefore, values are normalized to the individual value at experiment start for each experiment (X/X_t0_ = 100%). Additionally to blood count values between CT scans, values from the blood bag before (BB) and after the experiment (Control) are presented as the static control, also normalized to the experiment starting value (> 100%).

**Figure 3:**
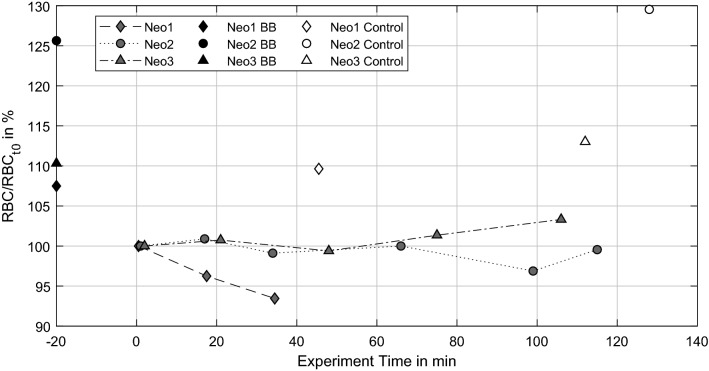
Red blood cell (RBC) count normalized to RBC (t0) over experiment time.

Red blood cell (RBC) count data is presented in Fig. 3. All static controls are in the range of ± 5%, which is in the range of the analyzer’s measurement error. Neo2 and Neo3 show only slight variations of a few percent from the beginning to the end of experiment, corresponding to the measurement error as well. Only Neo1 shows a decrease of RBCs from the experiment beginning onwards.

**Figure 4:**
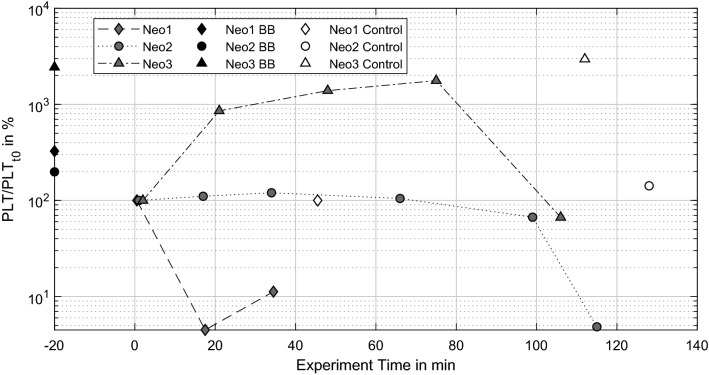
Platelet (PLT) count normalized to PLT (t0) over experiment.

Figure 4 shows the time course of platelet (PLT) numbers depicted in logarithmic scale. Control values of Neo2 and Neo3 remain stable over time; only the control of Neo1 shows a difference of 225% from blood bag to control measurement. Corresponding to the RBC decrease, PLTs of Neo1 steeply decrease within the first 20 min of experimentation and slightly increase towards the termination of the experiment. Values for Neo2 remain nearly constant up to 65 min, then slightly decrease over the next 35 min and finally reach 5% remaining PLTs after 115 min. In contrast, PLTs of Neo3 first increase up to 1766% over 80 min, followed by a steep decrease to 67% from the starting value to the experiment’s termination.

**Figure 5:**
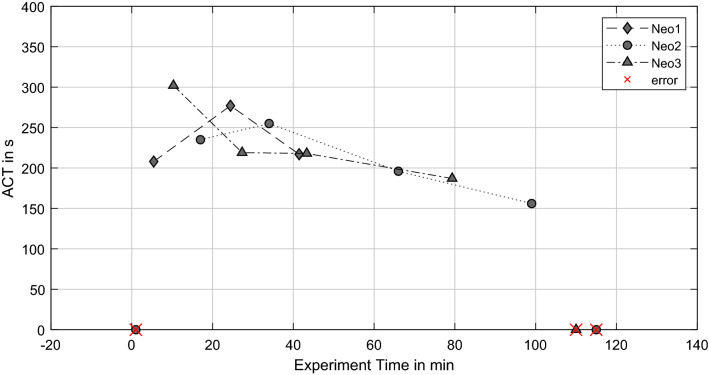
Activated clotting time (ACT) over the experiment time.

The course of ACT values over time is presented in Fig. 5. ACT decreases until the experiment termination for Neo2 and Neo3; only Neo1 shows a different behavior, which is in line with RBC and PLT values as well. Measurement errors that revealed no result are indicated with a red cross.

**Figure 6:**
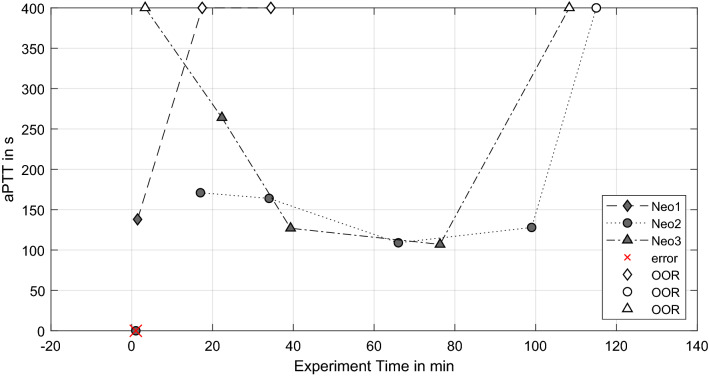
Activated partial thromboplastin time (aPTT) over the experiment time (OOR = out-of-range errors).

The upper limit of aPTT measurement was 400 s due to the utilized test cuvettes, indicated by an out-of-range-error (OOR) from the device. These values are set as 400 s (white symbols, see Fig. 6), whereas other incorrect measurements (e.g. insufficient sample volume) are indicated as “error”. Neo2 and Neo3 present decreasing aPTT values up to 30 min prior to the experiment termination. The last measurements before termination present a rapid aPTT increase of > 400 s. Neo1 reveals such an increase of aPTT as well, due to the shorter experiment time the rise of aPTT begins after the initial measurement.

### Experimental Flow Data

**Figure 7:**
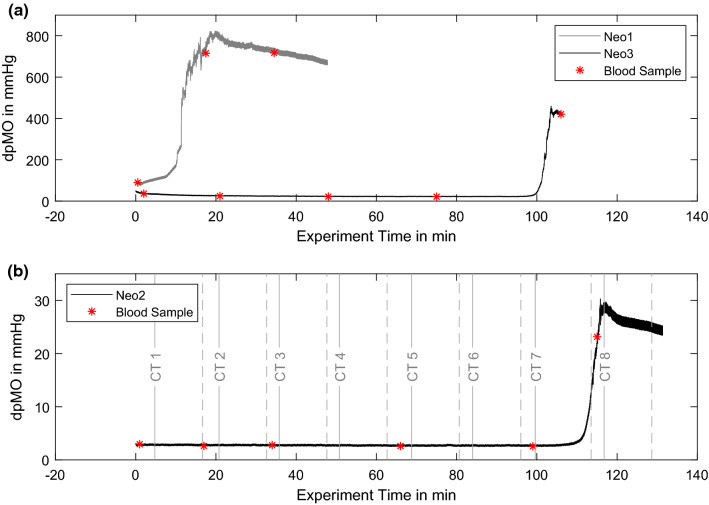
(a) Transmembrane pressure (dpMO) over the experiment time for Neo1 and Neo 3; (b) transmembrane pressure (dpMO) over the experiment time for Neo2.

The pressure gradient over the experiment time is presented in Fig. 7a for Neo1 & Neo3, and in Fig. 7b for Neo2 (different scaling). The time points of blood sampling are marked with red crosses and CT scanning periods are included for Neo2. However, all three oxygenators reveal a similar pressure course with a low plateau in the beginning and a rapid and steep increase towards the end and termination of the experiment. In accordance to the blood data, the Neo1 pressure gradient increases directly after the beginning of the experiment.

### X-Ray 3D Image Analysis

**Figure 8:**
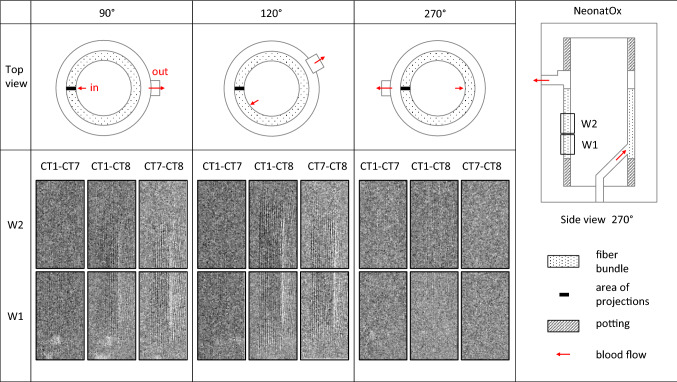
2D projections of the subtracted images CT1–CT7, CT1–CT8 and CT7–CT8 of analysis windows W1 and W2 between at different angles (grey values: − 200 to + 150).

Figure 8 illustrates the 2D projections of the subtracted images CT1–CT7, CT7–CT8 and CT1–CT8 for angles of 90°, 120° and 270° at two heights marked W1 and W2 of the membrane fiber bundle inside the Neo2 device. The grey values of the subtraction images are clipped to the range of − 200 to +150 without loss of information. Despite the CT alignment error of ± 1 pixel, two types of alterations were identified in the subtraction images.

- Fiber adjacent changes characterized by larger areas of parallel lines
- Local changes of one intensity defined by a larger number of coherent white or black pixel with high edge contrast (area size > 30 pixels) caused by thrombi (high pixel intensity = red thrombus, low pixel intensity = white thrombus) or moving air bubbles.

Comparison reveals the most prominent changes in grey scale in the subtraction image CT7–CT8. These observations indicate that the thrombus growth occurred between the last two scan CT7 and CT8, which agrees with the observed pressure increase, compare Fig. 7b.

**Figure 9:**
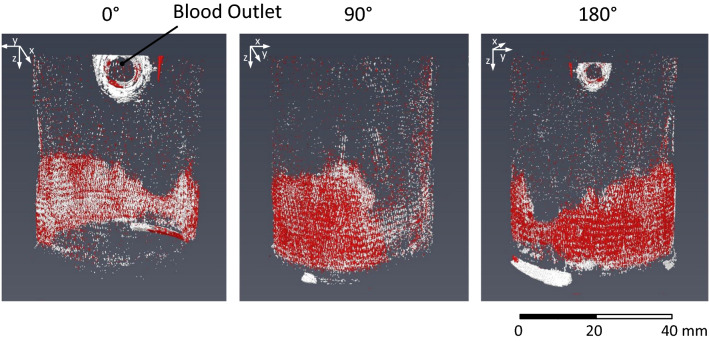
Volume rendering of the 3D subtraction data (CT1-minus-CT8) of Neo2 in 0°, 90°, and 180° rotation angle; higher and lower X-ray intensity in the subtraction data were segmented in red and white color, which relate to red and white thrombus formation, respectively.

Potential red and white clots were segmented in red and white, respectively, see Fig. 9. These regions are mainly present on the opposite site of the blood outlet at the punctual transition from inner core to the membrane fiber bundle.^2^ The intensity changes (red/white) at the outlet are located outside of the blood-filled outlet and can therefore be attributed to scatter and shifting due to moving tubing during image acquisition. All remaining intensity changes are located within the coiled fiber mat. Red and white areas are found in strand-like structures in fiber proximity. The red and white segmented volumes provide an indication of red (more RBCs) and white thrombus (less RBCs) formation, respectively.^5,6,19^

## Discussion

Transmembrane oxygenator thrombosis (MOT) is one of the main complications during extracorporeal membrane oxygenation (ECMO). Since MOT occurs time-independent and patient specific, ongoing research focuses on predicting thrombosis based on patient and oxygenator monitoring as well as reducing the thrombotic risk by optimizing oxygenator hemodynamics. Developing effective monitoring models requires profound knowledge of the flow dynamics and hemostatic processes in the oxygenator. Since current experimental methods are unable to image the formation of thrombosis due to the restricted optical accessibility of oxygenators, this study proposes a new method for *in-vitro* thrombus growth imaging in oxygenators via real-time computed tomography (CT) scanning in a dedicated setup.

Thrombus growth was successfully generated in three NeonatOx devices. All experiments were performed at a low anticoagulation dose of 2.5 USP mL^−1^ heparin and showed *in-vitro* thrombus formation characterized by a decrease in platelet count, activated clotting time (ACT), an increase in activated partial thromboplastin time (aPTT) as well as an increase in transmembrane pressure drop (dpMO) comparing values from the experiment start to experiment termination. Furthermore, a visual inspection of the blood loop and oxygenator showed adherent thrombi at tubing connectors, the blood reservoir, and the membrane fiber bundle of the oxygenators. For Neo1 we observed a steep increase in dpMO and a rapid decreased in RBC and PLT count within the first 20 min of the experiment. In combination with an increasing aPTT, this suggest an already advanced coagulation activation from blood management prior to the experiments. Even though the blood preparation protocol was identical for all experiments, an incident during blood withdrawal or a medical issue with the donor sheep might be the reason for the observed hypercoagulability. In the following, only the remaining two experiments are discussed.

The two experiments (Neo2 & Neo3) were terminated after 115 min and 105 min, respectively, due to a rapid increase in pressure drop in combination with a severe loss in PLT numbers. Such a loss of platelets is caused either by platelet activation since activated platelets are not detected as “platelets” by the blood analyzer, or by platelet consumption in formed thrombi. Both scenarios indicate ongoing coagulation within the test circuit, which is supported by constantly decreasing ACT and a rapid rise in aPTT by the end of the experiment. All values indicate an activation of the coagulation cascade and consequently a consumption of coagulation factors within the test loop. The final increase of aPTT in the light of the formed thrombi depicts the turning point in coagulation, where the majority of coagulation factors and components is already activated and included in the thrombi.

Irregularities in PLT count were obvious for Neo3 with a severe lower count of PLTs compared to the blood bag value at the start of the experiment but a recovery close to that value over 70 min. We assume that a temperature loss during flow loop filling triggered platelet activation, resulting in lower PLT numbers at the start of the test. A reversible platelet activation induced by low temperatures is a known phenomenon in literature.^23,32^ While the temperature rises, platelet activation is reversed and PLT count increases again. This might explain the increasing PLT numbers especially within the first 20 min of experimentation.

Clinical studies show controversial perspectives on dpMO-based MOT monitoring. While some studies demonstrate the benefit of MOT assessment by dpMO monitoring,^22,26^ Krivitski *et al.* describe dpMO monitoring alone as inaccurate, explaining that the increase in dpMO depends rather on thrombus location than size,^20^ which is supported by Lehle *et al.* stating that increasing dpMO is a rare device related indicator of MOT.^21^ Consequently, we presume that the large difference in maximum dpMO of both Neo2 and Neo3 experiments could be a result of different thrombus locations and geometries. We ascribe the final plateauing dpMO value to the development of adherent thrombi in the oxygenator. However, we want to point out that the accuracy of pressure measured in the current flow loop set up could have been compromised by thrombus formation at the pressure sensor, since examination of the flushed flow loop revealed adherent thrombi in the Luer-connectors next to the pressure sensors.

The NeonatOx device has a punctual transition from the inner core to the fiber bundle, which is located on the opposite site of the device’s blood outlet.^2^ PIV and CFD studies showed an inhomogeneous blood flow distribution inside the fiber bundle, especially in the area under the device blood outlet. Schlanstein presented evidence that this area is prone to thrombus formation.^29^ In our image data generated on experiment Neo2, however, we observed major thrombus formation directly at the area of the punctual transition from the inner core to the fiber bundle at the device inlet. We hypothesize that clots were formed in other areas of the blood loop and were flushed into the device. This hypothesis is supported by the visual inspection of the blood loop, which revealed thrombi not only in the test device but also at critical hotspots within the blood loop, i.e. connectors, pressure sensors and the blood bag. Also, the manual mixing of the blood reservoir during the experiments, which was incorporated into the protocol to minimize stagnation and cell sedimentation, may have ultimately led to a detachment of adherent thrombi. Contrary to previous work, we found no indication of thrombus formation in that part of the fiber bundle that is located below the outlet of the device.

We have shown that we can achieve a constant RBC count over the duration of the experiment, which does not lead to intensity changes caused by the blood itself. However, with the current resolution, the image subtraction method to identify thrombus growth via intensity changes cannot differentiate between white thrombus formation and air bubbles, which both show up as regions of lower X-ray attenuation in CT8. With our current effective pixel size of 135 *µ*m, we were also not able to detect initial thrombus growth in CT scans 1–7. However, it might be possible that thrombi smaller than this size were formed but not detected in the CT scans. Only a severe clotting event was observed in CT scan 8. Moreover, the temporal resolution needs improvement to detect thrombus growth in real-time rather than at discrete points in time.

This new method for thrombus detection within oxygenators can be further improved in the future by elimination of some limitations. Due to current spatial and temporal resolution limits, the CT visualization cannot distinguish between thrombi flushed into the oxygenator and thrombi that are initially formed within the test device. An optimization of the blood loop in terms of minimizing areas of stagnation (e.g. blood reservoir, sensors) and areas of high shear rates (e.g. connector edges) to prevent any thrombus growth except of device-related thrombi will strengthen the validity of the real-time CT visualization.

Platelet and especially pressure drop occurred within a short time of approximately 10 min. Extending this time frame to 30 min by a higher anticoagulation dose would allow for at least two CT scans during the period of the major oxygenator thrombus formation and thereby additional visual information about thrombus growth kinetics.

In future studies, human blood could be used instead of animal blood since the priming volume of the complete setup does not extend 160 mL and can therefore be provided within one single human blood donation. This would overcome possible difference in animal and human coagulation behavior and strengthen human MOT prediction.

Including gas exchange and therewith gas monitoring data into the test setup would provide additional technical parameters that might react to thrombosis in the test oxygenators. Thrombus formation and protein adsorption on the membrane surfaces hinders gas exchange performance of the complete device^9^ and might be an additional valuable prediction parameter when combined with imaging of thrombus growth. However, this would lead to a more complex test setup (e.g. for incorporation of a de-oxygenator) and thus lead to higher blood volumes in the test circuit.

Overall, we present blood and pressure data for n = 3 and CT data for n = 1 NeonatOx oxygenator devices as a first feasibility study (proof-of-concept) for this novel approach. Based on these first results, we are confident that the whole test setup is feasible to detect thrombus growth in oxygenators as a combination of clinically relevant parameters and real-time thrombus visualization. By taking additional monitoring parameters into account, the proposed *in-vitro* method might allow for distinct thrombus growth evaluation in membrane oxygenators and therewith contribute to better clinical oxygenator exchange protocols. In the long run, this will lead to an improved, safer and more specific patient care during ECMO treatment.
